# The Controversy of Surgical Intervention for Anal Canal Duplication in Children

**DOI:** 10.12669/pjms.36.6.1832

**Published:** 2020

**Authors:** Fatih Akova, Serdar Altinay, Emrah Aydin

**Affiliations:** 1 Dr. Fatih Akova, MD. Assistant Professor, Pediatric Surgery Department, Biruni University, Istanbul, Turkey. Pediatric Surgery Department, University of Health Science, Faculty of Medicine, Bagcilar Training & Research Hospital, Istanbul, Turkey; 2 Dr. Serdar Altinay, MD. Associate Professor, Pathology Department, University of Health Science Bakırköy, Dr. Sadi Konuk Training and Research Hospital, Istanbul, Turkey; 3 Dr. Emrah Aydin, MD. Assistant Professor, Pediatric Surgery Department, Koc University School of Medicine, Istanbul, Turkey

**Keywords:** Anal canal duplication, anorectal disease, presacral cystic mass, tailgut cyst

## Abstract

**Objective:**

Since the first definition of anal canal little has been discovered about the etiology of this rare condition. We present four asymptomatic cases of anal canal duplication with diverse clinical and surgical findings.

**Methods:**

A retrospective chart review was performed on four infants presenting with asymptomatic anal canal duplication, born between 2014 and 2016. Clinical characteristics and pathologic findings of patients either by radiological imaging or pathology were evaluated. The primary outcome measure was the complications.

**Results:**

All patients were followed-up with physical examination and ultrasound for a mean of 3.5±1.0 years, lastly seen at the beginning of 2018. The female to male ratio was 3:1. Duplicate anal canal length varied between 12-20mm, and two of the four patients had a presacral cystic mass confirmed as a tail gut cyst following surgery. At follow-up, none of the patients had developed symptoms related to anal canal duplication, regardless of whether they had surgical intervention.

**Conclusion:**

Though surgical management is the preferred treatment for anal canal duplication, it seems that patients who do not undergo surgery might remain free of symptoms, suggesting that surgical intervention may be unnecessary.

## INTRODUCTION

Since the anatomical definition of the anal canal by Symington in 1888, and the first published case of anal canal duplication (ACD) in a 65-year-old patient with colloid carcinoma, little has been discovered about the etiology of this condition.^1^-^3^ ACD is a very rare digestive duplication, with approximately 50 total cases reported in a PubMed research in the English literature. It is more commonly seen in females and mostly occurs at the 6 o’clock position relative to the anus. Although there is a general belief that unless resected, ACD may result in malignancy, only a single case has been reported in the literature to support this.^4^ Histologically, ACD is typically characterized by a combination of squamous epithelium, transitional epithelium, and smooth muscle cells.^5^ While some patients are asymptomatic, others suffer from a wide variety of symptoms, from pruritus to an intra-pelvic or intra-abdominal abscess or mass. These symptoms may not be representative of the disease progression or complexity. In this report, we present four asymptomatic cases of ACD with diverse clinical and surgical findings to further discuss on this controversy.

## METHODS

Following approval from the Institutional Review Board (Ref.no: 2018.234.IRB1.027 Dated October 16, 2018), a retrospective chart review was performed through hospital medical records between 2014 and 2016. Among all, four patients were found who presented to the outpatient clinic with a second opening posterior to the anal canal at the six o’clock position. The main focus of the study was to evaluate the clinical characteristics, preoperative diagnosis, and pathologic findings if resection was performed while the primary outcome was the complications during follow-up period.

## RESULTS

**Fig.1:**
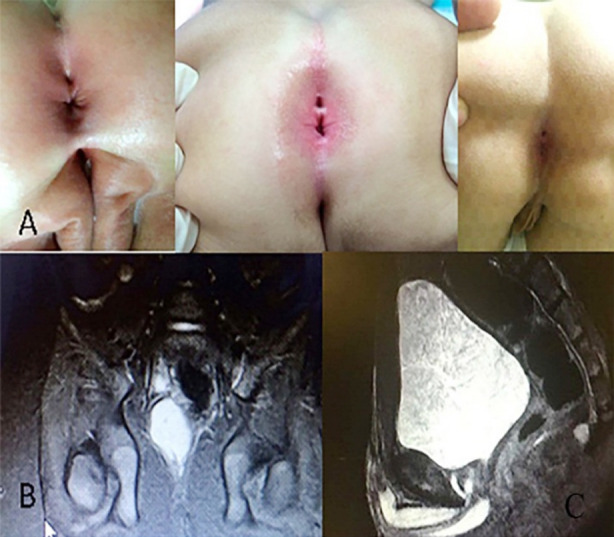
The perianal inspection demonstrates additional openings posterior to the anal orifice (A). Sagittal T1 weighted pelvic MRI demonstrates the presacral mass in patient 3 (B) and 4 (C).

Data was gathered from four patients over a two-year period. The female to male ratio was 3:1. Mean age at diagnosis was 41.25 months (range 3-144 months). All patients were asymptomatic when admitted to the hospital due to parental concern regarding the second perianal opening. An additional perianal orifice was observed in the midline at the six o’clock position in all four patients while they were supine during physical examination (Fig.1). Three patients were examined by ultrasound, while all four patients underwent magnetic resonance imaging (MRI). Two of the patients underwent a resection operation, while the rest could not be consented for surgery. All patients were followed-up with physical examination and ultrasound for a mean of 3.5±1.0 years, lastly seen in 2018.

In the diagnostic workup of these patients, three of the infants were examined by ultrasound, and all four were given an MRI (Table-I). Digestive communication could not be demonstrated in any of the patients. The length of the duplicate canal varied between patients, and the two longest canals were both accompanied by a presacral cystic mass, revealed as tailgut cyst following surgery.

**Table-I:** Characteristics of patients with anal duplications.

Patient	1	2	3	4
Age at diagnosis (months)	3	9	9	144
Gender	Female	Female	Female	Male
Length (mm)	12	13	20	20
Diagnosis	US/MRI	US/MRI	US/MRI	MRI
Pathology	None	None	Presacral cystic mass	Presacral cystic mass
Additional pathology	None	None	Tailgut cyst	Tailgut cyst
Surgical approach	Refused	Refused	PSARP	PSARP

US: Ultrasound, MRI: Magnetic Resonance Imaging, PSARP: Posterior Sagittal Ano-Recto Plasty.

**Fig.2:**
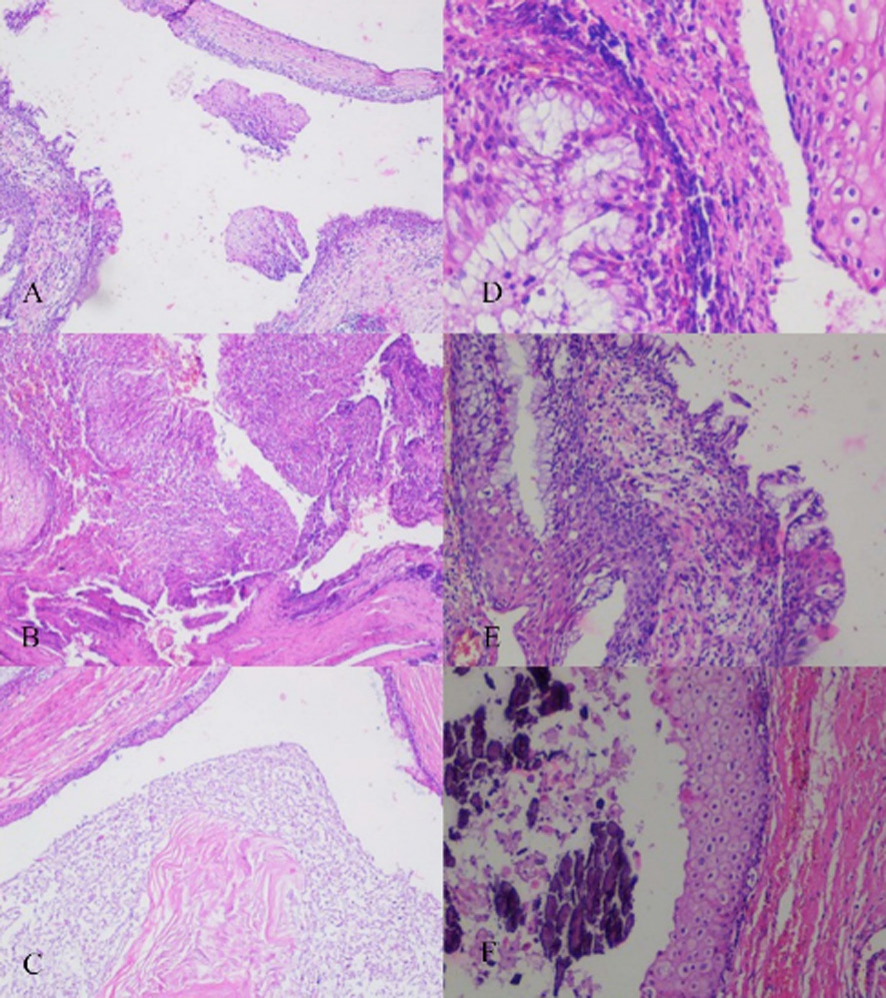
Posterior sagittal approach during surgical removal of cystic mass and removed pieces of duplicated anal canal.

**Fig.3:**
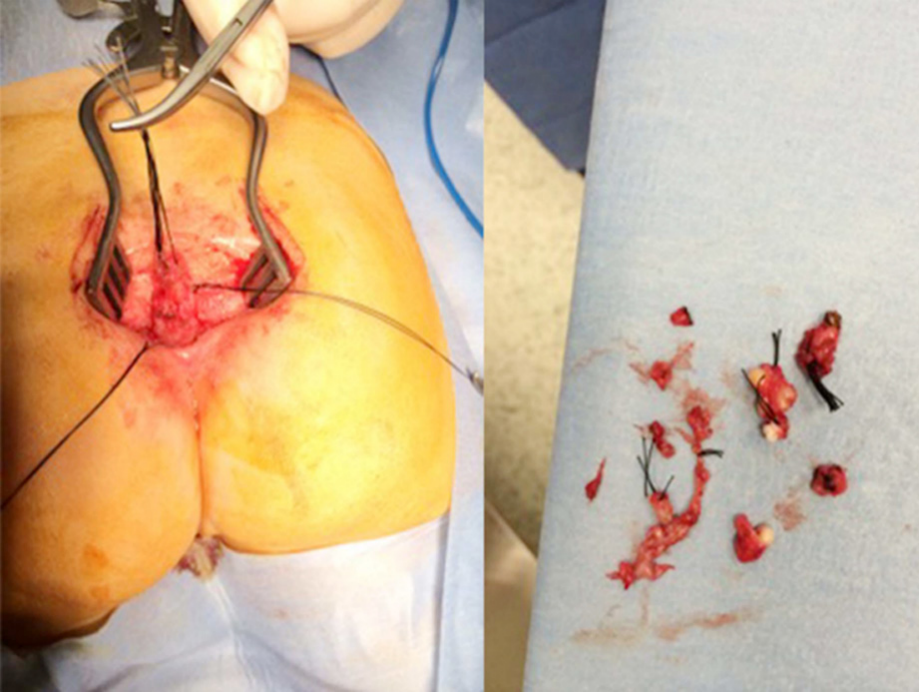
A) Connective tissue with mature stratified squamous epithelium and mild lymphocytic inflammation (40X H&E). B) Tissue with squamous epithelium that has lymphocytic inflammation (100X H&E). C) Cyst lined with squamous epithelium and containing eosinophilic keratinous material in its lumen (100X H&E). D) Cyst wall superficially lined with squamous epithelium and deeply lined with columnar, mucinous epithelium (400X H&E). E) Cyst lined with both squamous epithelium and columnar, mucinous epithelium and lymphocytic inflammation (100X H&E). F) Cyst lined with squamous epithelium and containing calcified amorphous material in its lumen. (200X H&E).

Only the patients with accompanying cystic mass could be consented for surgical management (Fig.2). A Posterior Sagittal Ano-Recto Plasty (PSARP) procedure was performed in both cases. Histopathological examination revealed mature stratified squamous epithelium with lymphocytic inflammation. In both patients, the mass had multiple lobules with a very thin wall and a shiny inner surface and was filled with a mucinous material (Fig.3).

In the two patients with a presacral cystic mass, surgery was successful and uneventful. Both patients were discharged from the hospital after a week and did not show symptoms related to their previous pathology when followed up with physical examination and ultrasound. The patients who could not be consented also showed no symptoms at follow-up.

## DISCUSSION

ACD is a rare condition in which a second perianal opening can be seen in addition to the normal orifice. The etiopathogenesis of the disease is still unclear, though it is speculated that there is either a developmental cloacal anomaly during fetal growth or disturbances in the recanalization process.^6^-^9^ Although the most alarming scenario is the development of cancer from ACD, only one case has so far been reported, which may have been sporadic.

There have been 50 cases of ACD reported in the literature to date. If the condition occurs as an isolated condition, it is mostly asymptomatic.^7^ Where patients report symptoms, the most common are abdominal pain, perianal pain, and constipation, all of which are quite nonspecific. On the other hand, Lisi et al. reported symptoms of mucous drainage in one patient and recurrent fistula in two patients out of 12 before the age of 35.^9^ It has been proposed that the probability of developing symptoms and complications increases with age. In this study, all patients were asymptomatic at presentation regardless of accompanying disorders. They were all admitted to the hospital due to parental concern regarding the second opening.

Cyst pathology is important in the differential diagnosis of duplication cysts. In the pathological examination, enteric cysts have less organized smooth muscle and do not contain nervous tissue.^10^ Specimens from the two surgical patients in this study exhibited organized smooth muscle and nervous plexus, identifying them as duplication cysts.

Although the more widely accepted management of anal duplication cysts is complete surgical excision through posterior sagittal approach, due to the potential for infection, increase in size and malignancy, the two patients whose families declined consent for the surgery have been free of symptoms so far.

ACD is rare, yet it poses a risk for recurrent infection and malignancy. A second opening posterior to the anal orifice leads to the suspicion of this condition. Surgery is required if there are associated conditions with ACD like meningocele, presacral cysts or masses and also for deep ACD. Conservative treatment may be considered in cases with isolated superficial ACD. Long term follow-up is required in cases managed conservatively

### Authors’ Contribution:

All authors made substantial contributions to conception, acquisition, analysis and interpretation of data.

## References

[1] Freeman DE (2012). Rectum and Anus. Equine Surg.

[2] Morgan CN, Thompson HR (1956). Surgical anatomy of the anal canal with special reference to the surgical importance of the internal sphincter and conjoint longitudinal muscle. Ann R Coll Surg Engl.

[3] Dukes CE, Galvin C (1956). Colloid carcinoma arising within fistulae in the anorectal region. Ann R Coll Surg Engl.

[4] Cheng LS, Courtier J, Mackenzie TC (2013). Anal duplication in a one-year-old girl. J Pediatr Surg Case Reports.

[5] Ochiai K, Umeda T, Murahashi O, Sugitoh T (2002). Anal-canal duplication in a 6-year-old child. Pediatr Surg Int.

[6] NarcI A, Dilek FH, Cetinkursun S (2010). Anal canal duplication. Eur J Pediatr.

[7] Koga H, Okazaki T, Kato Y, Lane GJ, Yamataka A (2010). Anal canal duplication:Experience at a single institution and literature review. Pediatr Surg Int.

[8] Ponson AE, Festen C (2001). Postanal sinus:Single or different etiologies?. Pediatr Surg Int.

[9] Lisi G, Illiceto MT, Rossi C, Broto JM, Jil-Vernet JM, Lelli Chiesa P (2006). Anal canal duplication:A retrospective analysis of 12 cases from two European pediatric surgical departments. Pediatr Surg Int.

[10] Stacey E, Stacey EM, Joel KG, Jason LH, Teri AL, Victor ER (2015). Mills. Chapter 33 Nonneoplastic intestinal diseases. 'Sternberg's Diagnostic Surgical Pathology'(sixth edition). Wolter Kluwer.

